# Mapping of single-copy genes by TSA-FISH in the codling moth, *Cydia pomonella*

**DOI:** 10.1186/1471-2156-15-S2-S15

**Published:** 2014-12-01

**Authors:** Leonela Z Carabajal Paladino, Petr Nguyen, Jindra Šíchová, František Marec

**Affiliations:** 1 Institute of Entomology, Biology Centre ASCR, Branišovská 31, 370 05 České Budějovice, Czech Republic; 2 Faculty of Science, University of South Bohemia, Branišovská 31, 370 05 České Budějovice, Czech Republic

**Keywords:** Acetylcholinesterase 1, Sex-linked gene, Chromosomes, Fluorescence in situ hybridization, Tyramide signal amplification, Tortricid moth

## Abstract

**Background:**

We work on the development of transgenic sexing strains in the codling moth, *Cydia pomonella *(Tortricidae), which would enable to produce male-only progeny for the population control of this pest using sterile insect technique (SIT). To facilitate this research, we have developed a number of cytogenetic and molecular tools, including a physical map of the codling moth Z chromosome using BAC-FISH (fluorescence in situ hybridization with bacterial artificial chromosome probes). However, chromosomal localization of unique, single-copy sequences such as a transgene cassette by conventional FISH remains challenging. In this study, we adapted a FISH protocol with tyramide signal amplification (TSA-FISH) for detection of single-copy genes in Lepidoptera. We tested the protocol with probes prepared from partial sequences of Z-linked genes in the codling moth.

**Results:**

Using a modified TSA-FISH protocol we successfully mapped a partial sequence of the *Acetylcholinesterase 1 *(*Ace-1*) gene to the Z chromosome and confirmed thus its Z-linkage. A subsequent combination of BAC-FISH with BAC probes containing anticipated neighbouring Z-linked genes and TSA-FISH with the *Ace-1 *probe allowed the integration of *Ace-1 *in the physical map of the codling moth Z chromosome. We also developed a two-colour TSA-FISH protocol which enabled us simultaneous localization of two Z-linked genes, *Ace-1 *and *Notch*, to the expected regions of the Z chromosome.

**Conclusions:**

We showed that TSA-FISH represents a reliable technique for physical mapping of genes on chromosomes of moths and butterflies. Our results suggest that this technique can be combined with BAC-FISH and in the future used for physical localization of transgene cassettes on chromosomes of transgenic lines in the codling moth or other lepidopteran species. Furthermore, the developed protocol for two-colour TSA-FISH might become a powerful tool for synteny mapping in non-model organisms.

## Background

Codling moth, *Cydia pomonella *(Linnaeus), has one of the worst reputation of all representatives of the microlepidopteran family Tortricidae, for it is virtually cosmopolitan pest that causes severe crop damage in pome fruit and walnut orchards [1-3]. The codling moth control has been mainly achieved by intensive use of broad spectrum chemical insecticides. However, their use led to the development of resistance and cross-resistance to many registered pesticides of various chemical classes [4-6]. Moreover, insecticide-induced disruption of natural control, i.e. elimination of non-target predator populations, may result in outbreaks of secondary pests such as aphids and phytophagous mites [7,8]. There are also concerns over environmental contamination and effect of insecticides on human health. These issues along with growing environmental awareness of the general public increase calls for efficient and sustainable pest control methods [9].

One such environment-friendly control method is the sterile insect technique (SIT), the effectiveness of which has been demonstrated in many insect pests [10]. The SIT has been successfully implemented as a part of area-wide integrated pest-management programmes for suppressing codling moth populations in British Columbia, Canada [11]. Since 1992, mass-reared moths have been sterilized by gamma radiation and released in the Okanagan region of British Columbia [12,13]. The sterile males mate with wild females but produce no offspring due to the radiation-induced dominant lethal mutations transferred by sperm [14]. Elimination of codling moth infestation along with a drop in the use of insecticides in most treated orchards of British Columbia has increased interest in the expansion of codling moth SIT to other countries [15]. The codling moth SIT relies on bisexual releases, but there are reasons to believe that male-only releases would bring significant improvement to this technology as it has been demonstrated in the medfly, *Ceratitis capitata*, where the release of irradiated males only is severalfold more efficient in inducing sterility in wild insect populations than the release of both sexes [16-19]. However, an efficient technology of sex separation that would be easily applicable under mass rearing conditions has not yet been developed in *C. pomonella*. Therefore, a new approach for genetic sexing in the codling moth has been proposed. It is based on the development of a transgenic *C. pomonella *strain with females carrying a dominant conditional lethal mutation (DCLM) in the female-specific W chromosome. Under restrictive conditions all female progeny would be eliminated due to the presence of the W chromosome bearing the DCLM transgene, whereas non-transgenic males would survive and could be irradiated and released upon emergence [20]. A dominant cold sensitive mutant allele of the *Notch *gene, originally isolated in *Drosophila melanogaster*, has been chosen as a suitable DCLM [21], and the codling moth transgenesis is currently under way [22].

To facilitate the development of genetic sexing strains, the codling moth genome organization has been extensively studied with emphasis on its sex chromosomes and their molecular differentiation. It was shown that the karyotype of *C. pomonella *consists of 2n = 56 holokinetic chromosomes, i.e. chromosomes lacking localized centromere [23], which seems to represent the modal chromosome number in the tortricid subfamily Olethreutinae [24] to which the codling moth belongs. The largest chromosome pair corresponding to the W and Z sex chromosomes in females was further probed by means of genomic in situ hybridization, comparative genomic hybridization, and W-specific painting probes. The codling moth W chromosome was shown to be highly differentiated from the Z chromosome at the molecular level and composed predominantly of repetitive sequences [23,25]. W-derived female specific molecular markers were also developed and successfully used to identify the sex in the early developmental stages of the codling moth [22]. Furthermore, comparative physical mapping of the Z sex chromosome was performed in the codling moth using fluorescence in situ hybridization (FISH) with probes derived from bacterial artificial chromosome (BAC) clones. The mapping of genes using BAC-FISH revealed that the Z chromosome of this species arose by a fusion between an ancestral Z sex chromosome and an autosome corresponding to chromosome 15 in the silkworm (*Bombyx mori*) reference genome. Interestingly, the silkworm chromosome 15 harbours the *ABC transporter C2 *(*ABCC2*) and *Acetylcholinesterase 1 *(*Ace-1*) genes, which confer resistance to *Bacillus thuringiensis *toxin Cry1Ab and insensitivity to organophosphate and carbamate insecticides, respectively. In addition, the *Notch *gene, whose mutant form is proposed for germ-line transformation of the codling moth, is also located on chromosome 15 in the silkworm. While *ABCC2 *and *Notch *were mapped directly to the codling moth Z chromosome by BAC-FISH, no BAC clone was available in case of *Ace-1*, and its sex-linkage was inferred from a comparison of male and female gene dose by quantitative PCR (qPCR) [26].

Both physical map of the codling moth Z chromosome and W-derived female specific molecular markers can be used for physical mapping of transgenes in genetic sexing strains once available. However, chromosomal localization of unique, single-copy sequences such as a transgene cassette remains challenging, since threshold for routine detection by conventional FISH protocols ranges between 5 to 10 kbp [27,28]. In this study, we adapted a FISH protocol involving enzyme mediated deposition of fluorophore-labelled tyramide, the so-called tyramide signal amplification (TSA-FISH) previously used in *Xenopus tropicalis *[29-33], for detection of single-copy genes in Lepidoptera. We successfully localized the *Ace-1 *gene on both mitotic and meiotic chromosomes of the codling moth and confirmed its sex-linkage. Combination of BAC-FISH with reprobing protocol and TSA-FISH allowed us to add *Ace-1 *to the existing physical map of the codling moth Z chromosome. Furthermore, we have developed a protocol for two-colour TSA-FISH that may substantially facilitate comparative mapping in non-model organisms.

## Methods

### Chromosome preparations

We used *Cydia pomonella *specimens of a laboratory wild-type strain referred to as Krym-61 (for its origin, diet, and rearing conditions, see [23]). Spread chromosome preparations were made from male gonads of 4-5th instar larvae as described by Mediouni et al. [34]. Briefly, testes were dissected in a physiological solution, pre-treated for 10 min in a hypotonic solution (0.075 M KCl) and fixed in Carnoy fixative (ethanol/chloroform/acetic acid, 6:3:1) for 15 min. Tissue was subsequently transferred into a drop of 60% acetic acid, dissociated with tungsten needles and spread on the slide using a heating plate at 45°C. The preparations were passed through a graded ethanol series (70, 80, and 100%; 30 s each) and stored at -20°C until further use.

### FISH with bacterial artificial chromosomes (BAC-FISH)

BAC clones 40B18 and 12O03 containing the genes *Nanchung *(*Nan*) and *Ribosomal protein P0 *(*RpP0*), respectively, were obtained from the codling moth BAC library constructed by GENEfinder Genomic Resource Laboratory (Texas A&M University, College Station, TX, USA) (for details, see [26]). BAC-DNA was extracted using Qiagen Plasmid Midi Kit (Qiagen, Düsseldorf, Germany) and labelled by Cy3-dUTP (GE Healthcare, Buckinghamshire, UK) or ChromaTide Fluorescein-12-dUTP (Invitrogen, Paisley, UK) using a Nick Translation Kit (Abbott Molecular, Des Plaines, IL, USA) as described by Nguyen et al. [26]. Two-colour BAC-FISH was performed according to Yoshido et al. [35]. The probe cocktail for one slide (10 μL; 50% deionized formamide and 10% dextran sulphate in 2x SSC buffer) contained 160-450 ng of each labelled BAC probe, 3 µg of unlabelled sonicated genomic DNA used as a species-specific competitor and 25 µg of sonicated salmon sperm DNA (Sigma-Aldrich, St. Louis, MO, USA). Reprobing protocol was adapted from Shibata et al. [36].

### FISH with tyramide signal amplification (TSA-FISH)

Partial coding sequences of the *Acetylcholinesterase 1 *(*Ace-1*) (~1300 bp) and *Notch *(~1300 bp) genes were amplified by PCR as described in Nguyen et al. [26]. The PCR products were extracted from the gel using Wizard^® ^SV Gel and PCR Clean-Up System (Promega, Madison, WI, USA) and cloned into a pGEM^®^-T Easy Vector (Promega). Plasmids were isolated from the bacteria using a NucleoSpin^® ^Plasmid kit (Macherey-Nagel, Düren, Germany) and used as a template for PCR reamplification of the *Ace-1 *and *Notch *genes. Reamplified fragments purified by Wizard^® ^SV Gel and PCR Clean-Up System (Promega) were labelled for 1 h 45 min at 15°C with dinitrophenol-11-dUTP (DNP) (PerkinElmer, Waltham, MA, USA) or digoxigenin-11-dUTP alkali labile (DIG) (Roche Diagnostics, Mannheim, Germany) using a Nick Translation Kit (Abbot Molecular). For DIG-labelling, the same dNTP concentrations as for labelling of BAC DNA were used (see [26]). In case of DNP-labelling, manufacturer's recommendation was followed. Obtained probes were purified with illustra ProbeQuant G-50 Micro Columns (GE Healthcare).

The TSA-FISH experiments were performed according to Krylov et al. [30] with modifications. Slides were dehydrated in an ethanol series (70, 80, and 100%; 30 s each) and pre-treated with 50 µg/mL pepsin in 0.01 M HCl at 37°C for 5 min, 2% formaldehyde freshly prepared from paraformadehyde at room temperature (RT) for 10 min, 1% H_2_O_2 _in PBS at RT for 30 min, and 100 µg/mL RNase A in PBS at 37°C for 1 hour. After each pre-treatment, the slides were washed three times in PBS at RT for 5 min each. After the last washing, the slides were again dehydrated in an ethanol series (70, 80, and 100%; 3 min each). The chromosomes were hybridized with 50 μL of probe cocktail containing 50% deionized formamide, 10% dextran sulphate, and 40 ng of probe in 2x SSC. The probe cocktail was applied to chromosome preparation, covered with cover slip and denatured at 70°C for 5 min. The slides were then placed in a humid chamber. Hybridization was carried out at 37°C for 12-16 h.

After hybridization, the slides were washed three times in 50% formamide in 2x SSC at 42°C for 5 min each, three times in 2x SSC at RT for 5 min each, and one time in TNT buffer (0.1 M Tris-HCl pH 7.5, 0.15 M NaCl, 0.05% Tween-20) at RT for 5 min. The blocking of slides was carried out with 1 mL of TNB buffer per slide (0.1 M Tris-HCl pH 7.5, 0.15 M NaCl, 0.5% Blocking Reagent; PerkinElmer) at RT for 30 min. The excess of buffer was poured off and the slides were incubated at RT for one hour with 1 mL of anti-DIG-POD (Roche Diagnostics) or anti-DNP-HRP conjugate (PerkinElmer) diluted 1:1000 in TNB buffer. The slides were washed three times in TNT buffer for 5 min each. Tyramide amplification was carried out using the TSA Plus Fluorescence Kit with either Cy3 or fluorescein derivative (PerkinElmer) according to the instructions of the manufacturer. One hundred µL of the tyramide working solution was applied to a slide and incubated in a humid chamber for 15 min. The slides were then washed three times in TNT buffer for 5 min each and one time in 1% Kodak PhotoFlo (Sigma-Aldrich) in H_2_O for 1 min. The preparations were mounted in antifade based on DABCO (Sigma-Aldrich) (for composition, see [34]) containing 0.5 mg/mL DAPI (4′,6-diamidino-2-phenylindole) (Sigma-Aldrich).

In the case of two-colour TSA-FISH, probes were detected sequentially as shown e.g. by van Gijlswijk et al. [37] and Denkers et al. [38]. After last washing with TNT buffer, antibody conjugated peroxidase from the previous reaction was quenched with 1% H_2_O_2 _in PBS at RT for 30 min. The slides were washed three times in TNT buffer at RT for 5 min each, and the blocking, antibody incubation, and signal amplification steps were repeated.

### Microscopy and image processing

Chromosome preparations were observed in a Zeiss Axioplan 2 microscope (Carl Zeiss, Jena, Germany) equipped with appropriate fluorescence filter sets. Black-and-white images were captured with an Olympus CCD monochrome camera XM10 equipped with cellSens 1.9 digital imaging software (Olympus Europa Holding, Hamburg, Germany). The images were pseudocoloured and superimposed with Adobe Photoshop 10.0.1 (Adobe Systems, San Jose, CA, USA). Image analysis was performed using freeware ImageJ (National Institutes of Health, Bethesda, MD, USA).

## Results

We adapted the protocol of TSA-FISH, i.e. fluorescence in situ hybridization coupled with tyramide signal amplification, originally customized for the localization of genes on large mitotic metaphase chromosomes of the frog *Xenopus tropicalis *[30], for lepidopteran chromosomes. However, holokinetic chromosomes of Lepidoptera are unfavourable for cytogenetic analyses in mitotic metaphase due to their small size, the dot-like appearance, and the absence of any morphological landmarks such as localized centromeres. Therefore, much longer meiotic bivalents in the pachytene stage are employed for chromosome research and especially for FISH mapping (reviewed in [39-41]). Since pachytene bivalents are less resistant than compact mitotic chromosomes to particular FISH procedures due to their specific morphology, the following modifications of the Krylov et al. protocol [30] were applied. The duration of the slide pre-treatment with pepsin and formaldehyde was shortened in order to preserve the structure of the pachytene chromosomes, and an extra pre-treatment with RNase A was included to reduce background noise. Dextran sulphate was added to the hybridization mixture to increase the effective probe concentration and accelerate the hybridization rate, thus obtaining better quality signals. Finally, denaturation of the chromosomes and the probes was carried out at 70°C instead of 80°C to reduce its negative effect on the chromosome morphology.

**Figure 1:**
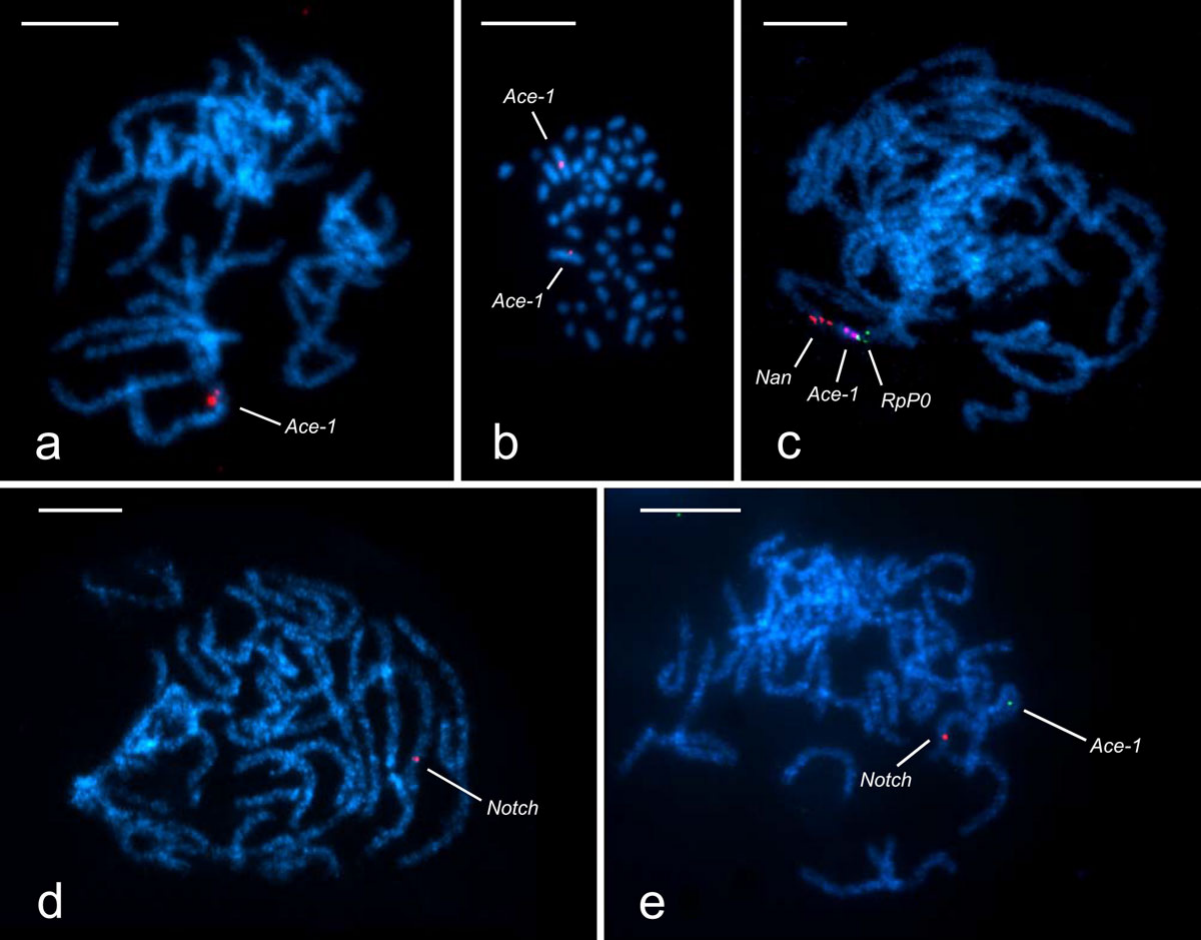
**Mapping of genes on chromosome preparations from testes of the codling moth, *Cydia pomonella***. Chromosomes were counterstained by DAPI (light blue). Hybridization signals (green, red, and violet) mark the physical position of genes under study (*Ace-1, Nan, RpP0*, and *Notch*). (a) *Ace-1 *localized on one bivalent in the pachytene spermatocyte complement by TSA-FISH; note two discrete but unbalanced (one large and one small) hybridization signals, each representing the *Ace-1 *locus on one homologous chromosome. (b) TSA-FISH with the *Ace-1 *probe revealed a nearly middle position of the *Ace-1 *locus on two largest elements of male mitotic metaphase corresponding to the Z chromosomes. (c) Two Z-linked genes, *RpP0 *and *Nan*, were mapped to the Z chromosome bivalent in the pachytene nucleus by BAC-FISH. Subsequent TSA-FISH localized the *Ace-1 *gene between these two markers. (d) The *Notch *gene mapped to a subterminal region of the Z chromosome bivalent in pachytene chromosomes by TSA-FISH. (e) Two single copy genes, *Ace-1 *and *Notch*, mapped to the Z chromosome bivalent in pachytene chromosomes by two-colour TSA-FISH. (Scale bar: 10 µm.)

TSA-FISH allowed the detection of unique sequences as short as ~1300 bp on both mitotic and meiotic chromosomes of the codling moth. As previously reported, this technique provided unbalanced signals, i.e. signals, which often differed in their intensity between homologous loci or signals that were observed only on one of the homologous chromosomes (Figure 1a, b, d and Figure 1e, respectively), most likely due to chromatin structure [28,42]. However, this obstacle is circumvented as pachytene bivalents formed by paired homologues are usually used for chromosome mapping in Lepidoptera [43-48]. The signal frequency, defined as a proportion of chromosome complements bearing at least one signal per bivalent, was higher than 60%. TSA-FISH thus represents a reliable means for detection of unique sequences on lepidopteran chromosomes.

**Figure 2:**
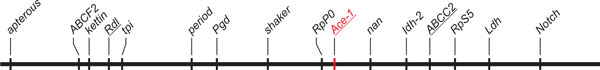
**Diagram integrating *Ace-1 *into a physical map of the codling moth Z chromosome**. The gene-based physical map was adopted from Nguyen et al. [26]. The mean relative position of *Ace-1 *was calculated from physical distances between *Ace-1 *and *RpP0 *hybridization signals related to physical distances between *RpP0 *and *Nan *in 10 ZZ bivalents. Major genes conferring insecticide resistance are underlined, *Ace-1 *is in red.

The probe derived from partial coding sequence of the *Acetylcholinesterase 1 *(*Ace-1*) gene provided a clear and intense hybridization signal localized nearly in the middle of a male pachytene bivalent (Figure 1a). Chromosomal position of the *Ace-1 *was also confirmed by TSA-FISH performed on male mitotic chromosomes, which mapped *Ace-1 *to an interstitial region of the two largest elements in the codling moth karyotype (Figure 1b) identified previously as the Z sex chromosomes [23,26]. Comparison of the physical map of the *Cydia pomonella *Z chromosome with a reference genome of the silkworm *Bombyx mori *[26] suggests that *Ace-1 *is located between two anchoring loci, *Ribosomal protein P0 *(*RpP0*) and *Nanchung *(*Nan*). In order to test this prediction we combined BAC-FISH and TSA-FISH in two subsequent steps. First, BAC-derived probes containing *RpP0 *and *Nan *were hybridized to male pachytene chromosomes of the codling moth. As expected, both markers were localized interstitially on a single pachytene bivalent (Figure 1c). Then the slides were stripped and reprobed using TSA-FISH protocol. The *Ace-1 *gene mapped between the two anchoring genes adjacent to *RpP0 *(Figure 1c). Physical distances between the genes were measured using ImageJ and relative position of *Ace-1 *was calculated, which allowed its integration in the physical map of the codling moth Z chromosome (Figure 2).

TSA-FISH could, in theory, be used for simultaneous detection of two markers, as both signals are expected to be present in about one third of nuclei due to high signal frequencies. For the two-colour TSA-FISH, sequential detection of probes labelled by DIG and DNP was adopted [37,38]. The *Notch *gene located in a sub-terminal region of the codling moth Z chromosome [26] (Figure 1d) was used along with *Ace-1 *to demonstrate feasibility of this approach. Two-colour TSA-FISH indeed mapped both genes to the Z chromosome bivalent in pachytene spermatocytes of the codling moth (Figure 1e). However, a substantial drop in the frequency of hybridization signals was observed after the second detection round of TSA-FISH. Since signal frequencies do not differ for particular haptens or fluorochromes used (not shown), the observed decrease in TSA-FISH efficiency was probably due to damage caused to hapten molecules while quenching the peroxidase activity with H_2_O_2 _after the first detection round.

## Discussion

A sequenced genome of the silkworm *Bombyx mori *[49-51] integrated with its BAC-based linkage map [52] spurred comparative genomic studies in Lepidoptera. BAC clones have been widely used for testing synteny of genes between silkworm and other lepidopteran species by BAC-FISH [26,35,43,44,47,48] as they provide reliable hybridization signals due to the large insert size ranging from 100 to 200 kbp [44,53]. Yet the BAC libraries are available only for a handful of species, and their construction, handling and screening remains rather expensive and laborious. Genes have also been physically mapped by conventional FISH with probes derived from cDNA (cDNA-FISH). Although this technique has been routinely utilized in vertebrates (e.g. [54-57]), in Lepidoptera it was used only for the identification of chromosomes involved in the evolution of the neo-sex chromosome systems in populations of wild silkmoths, *Samia cynthia *ssp. [45]. However, Vicoso et al. [58] found inconsistencies between results of cDNA-FISH and actual genomic data in snakes, which questioned this approach. Recently, up to 40 kbp long fosmid clones got in focus. Since fosmid libraries are easier to construct, they are expected to facilitate comparative gene mapping in Lepidoptera [46,47,59]. Since no genomic libraries are needed for fluorescence in situ hybridization with tyramide signal amplification (TSA-FISH) it has a potential to further facilitate cost-effective comparative genomic studies in non-model species.

The TSA-FISH protocol employed in this study, adapted to fine structures of meiotic chromosomes of Lepidoptera, allowed us to overcome the detection constraints of conventional FISH and visualize unique, about 1300 bp long sequences (Figure 1a-e). With TSA-FISH applied to chromosomes of the codling moth, we achieved hybridization signal frequencies of more than 60% at fluorescence intensities comparable to those of BAC clones (Figure 1c). Providing that an average size of a codling moth BAC clone insert is about 140 kbp [26], sensitivity of the probe detection by TSA-FISH was presumably enhanced about 100 times, reaching the upper range reported previously [60,61].

The potential of TSA-FISH was clearly demonstrated by mapping the *Acetylcholinesterase 1* gene (*Ace-1*) on the codling moth Z chromosome (Figure 1b,c,e). Nguyen et al. [26] physically mapped the codling moth Z chromosome and found out that it arose from a fusion between an ancestral Z sex chromosome and an autosome corresponding to the silkworm chromosome 15. The *B. mori *chromosome 15 was shown to harbour several genes conferring resistance to chemical and biological insecticides, namely *Resistance to dieldrin *(*Rdl*)*, ABC transporter C2 *(*ABCC2*) and *Ace-1*. The *ABCC2 *and *Rdl *genes were both unambiguously mapped to the codling moth Z chromosome by means of BAC-FISH. However, no BAC clone was available for the *Ace-1 *gene, which was assigned to the Z sex chromosome indirectly by quantitative PCR (qPCR) analysis of male and female gene dose. Yet uncertainty remained as it was shown that a chromosomal region adjacent to *Ace-1 *was translocated to an autosome in a common ancestor of the subfamily Olethreutinae [26]. TSA-FISH results confirmed the conclusions of qPCR analysis and localized *Ace-1 *to the expected chromosomal locus delimited by two anchoring genes, *Ribosomal protein P0 *and *Nanchung *(Figure 1c, Figure 2).

Nguyen et al. [26] argued that Z-linked mutations conferring resistance can be fixed faster in a pest population due to their hemizygosity in the females (cf. [62]). Although *ABCC2 *mutations conferring resistance to *Bacillus thuringiensis *toxin Cry1Ab were indeed reported to be recessive [63-65], the insensitivity conferred by *Ace-1 *is supposed to be semidominant to dominant [66]. However, Bourguet et al. [67] analyzed *Ace *alleles conferring insecticide resistance in mosquito strains and found out that dominance levels differ between strains, ranging from recessiveness to dominance. The authors explained the recessiveness of *Ace*-conferred resistance by activity of insensitive Ace. When Ace activity is low, heterozygotes (A^R^A^S^, where A stands for autosome) do not have sufficient amount of insensitive Ace compared to homozygotes (A^R^A^R^), and thus display a lower tolerance to insecticide. In tortricids, there would be no difference in activity of insensitive Ace-1 between heterozygous males (Z^R^Z^S^) and hemizygous females (Z^R^W) due to the sex-linkage of the *Ace-1 *gene and absence of global dosage compensation ([68], but see [69,70]). Therefore, there is seemingly no way to fix a recessive mutation conferring Ace-1 insensitivity. Yet Ace-1 insensitivity was reported to be sex-linked and recessive in another tortricid pest, the oriental fruit moth *Grapholita molesta *[71]. Kanga et al. [71] stated that the "inheritance of the *AChE *factor was either recessive or incompletely dominant depending on the direction of the cross". However, the authors misinterpreted the data since the *Ace-1 *inhibition in the F_1 _progeny from (R♀ × S♂) crosses reflected its sex-linkage rather than its incomplete dominance as F_1 _females from this cross are susceptible because they inherit the Z chromosome from susceptible male and W chromosome from resistant female. According to the results of (S♀ × R♂) cross the Ace-1 insensitivity is recessive. As hypothesised by Bourguet et al. [67] , recessiveness of Ace-1 insensitivity was most likely allowed by a female specific modifier compensating for a lower dosage of *Ace-1*, which most likely evolved in *G. molesta *as suggested by similar Ace-1 activity between males and females of both susceptible and resistant strains [71].

To conclude, TSA-FISH represents a reliable technique for physical mapping of genes on chromosomes of moths and butterflies. In future, this technique can be combined with BAC-FISH protocol (Figure 1c) and used for physical localization of transgene cassettes, such as that containing a dominant cold sensitive mutant allele of the *Notch *gene on sex chromosomes of transgenic lines of the codling moth or other lepidopteran species, if available. Furthermore, a developed protocol for two-colour TSA-FISH represents a feasible approach for comparative mapping of genes on the holokinetic chromosomes of moths and butterflies. After necessary optimization, the two-colour TSA-FISH might become a powerful tool which will allow cost-effective synteny mapping in non-model organisms.

## List of abbreviations used

*ABCC2*: *ABC transporter C2 *gene; *Ace-1*: *Acetylcholinesterase 1 *gene; BAC: bacterial artificial chromosome; BAC-FISH: fluorescence in situ hybridization with bacterial artificial chromosome probes; DCLM: dominant conditional lethal mutation; FISH: fluorescence in situ hybridization; *Nan*: *Nanchung *gene; PCR: polymerase chain reaction; qPCR: quantitative polymerase chain reaction; *Rdl*: *Resistance to dieldrin *gene; *RpP0*: *Ribosomal protein P0 *gene; RT: room temperature; SIT: sterile insect technique; TSA: tyramide signal amplification; TSA-FISH: fluorescence in situ hybridization with tyramide signal amplification.

## Competing interests

The authors declare that they have no competing interests.

## Authors' contributions

LZCP adapted the TSA-FISH protocol to lepidopteran chromosomes. LZCP and PN performed the experiments and analyzed the data. JŠ participated in preparation of chromosome spreads, FISH probes, and reagents. LZCP, PN and FM conceived this work and wrote the paper. All authors read and approved the final manuscript.
